# Peeling paint dermatosis

**DOI:** 10.11604/pamj.2024.47.76.42420

**Published:** 2024-02-20

**Authors:** Ashwin Karnan

**Affiliations:** 1 Department of Respiratory Medicine, Jawaharlal Nehru Medical College, Datta Meghe Institute of Higher Education and Research, Sawangi (Meghe), Wardha, Maharashtra, India

**Keywords:** Malnutrition, oedema, protein, kwashiorkor

## Image in medicine

A 4-month-old infant presented with complaints of inability to take feeds, peeling skin all over the body, with a history of loose stools two weeks back. There was no significant past or birth history. On examination, the infant was irritable, with generalized oedema present, dehydrated, weight for height in 62^nd^ percentile, pulse rate 130 beats/minute, respiratory rate 32 breaths/minute, blood pressure 80/60 mmhg, reduced breath sounds on auscultation. Chest X-ray done. Relevant blood investigations were done which showed anaemia, hypoalbuminemia, and dyselectrolytemia. A diagnosis of flaky paint dermatosis was made. The infant was treated with intravenous fluids, total parenteral nutrition, intravenous albumin, multivitamins, and other supportive medications. The infant improved clinically after 8 days, discharged, and the mother was advised to continue exclusive breastfeeding. Protein-energy malnutrition occurs due to inadequate protein and calories in the body, either due to increased need or due to reduced intake. Kwashiorkor is the less common type with an incidence of 3 per 1000 person months in the age group of 2-3 years. Clinically it is characterized by irritability, generalized oedema, distended abdomen, organomegaly, and dermatosis. Skin changes include dry skin which progresses to keratosis and hyperpigmentation. Gradually the fragile skin peels away exposing the hypopigmentation below. Treatment is protein and calorie supplementation and gradual introduction to enteral feeds.

**Figure 1:**
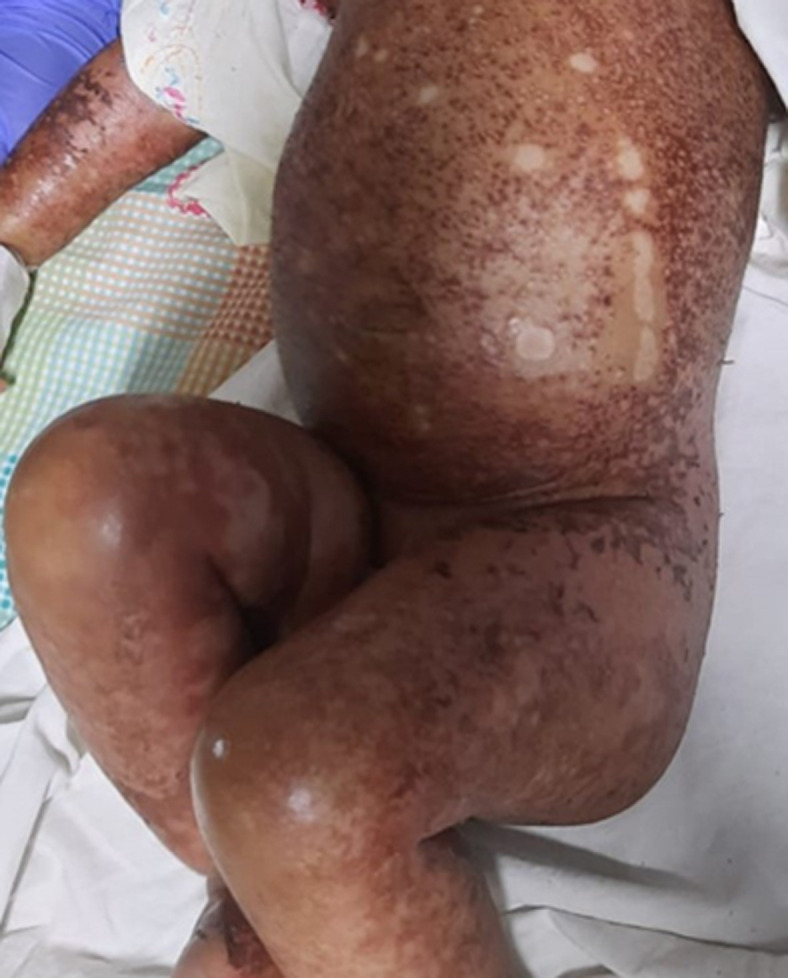
generalised hyperpigmented scaly lesions with areas of hypopigmentation

